# Spread of the *bla*_OXA-48_/IncL Plasmid within and between Dogs in City Parks, France

**DOI:** 10.1128/spectrum.00403-22

**Published:** 2022-05-31

**Authors:** Marisa Haenni, Véronique Métayer, Agnese Lupo, Antoine Drapeau, Jean-Yves Madec

**Affiliations:** a Unité Antibiorésistance et Virulence Bactériennes, Université de Lyon–ANSES laboratoire de Lyon, Lyon, France; Institute of Biomedical Sciences, Universidade de São Paulo

**Keywords:** OXA-48, dog, transmission

## Abstract

The *bla*_OXA-48_/IncL plasmid is increasingly reported in dogs, even in the absence of carbapenem use in animals. In this study, we witnessed the spread of this plasmid within and between dogs sharing the same relaxing area. This indicates a very dynamic situation where carbapenem resistance can be transmitted between dogs and expanded in the dogs’ gut. As a consequence, picking up dog feces may lower both this dynamic and the global antimicrobial resistance burden.

**IMPORTANCE** The use of carbapenems in animals is forbidden in France due to their critical importance to treat human diseases. Nevertheless, *bla*_OXA-48_-producing Enterobacterales were sporadically recovered in cats and dogs, most likely as a spill over from the human reservoir. This study highlights the rapid spread of *bla*_OXA-48_ once transmitted to dogs, suggesting that companion animals can play a role in the transmission routes of carbapenemase genes.

## OBSERVATION

Carbapenems (CPs) are antibiotics of critical importance for human health but not registered for use in animals, contrary to extended-spectrum cephalosporins (ESCs) which are widely used. ESC-resistant (ESC-R) and, more surprisingly, CP-resistant (CP-R) Enterobacterales have been reported in diverse animal species worldwide, from wild birds to food-producing animals (1, 2). Such resistant isolates were also found in Europe where regulatory measures and numerous incentives toward prudent use of antibiotics have been implemented in the animal sector. CP-R isolates are especially found in companion animals, in which advanced therapeutics and intensive care, including incidental off-label administration of CPs, are most developed. To date, *bla*_OXA-48_ has been by far the most frequently reported CP-encoding gene in pets in Europe. First identified in cats and dogs in 2013 in Germany (3), and in 2015 in a dog in France (4), its source has often been attributed to contact with humans, even though its dynamic of transmission has not been deciphered yet. In this study, we investigated whether dogs’ feces in urban public areas may play a role in the spread of ESC, and, more importantly, CP-producing Enterobacterales.

In June 2018, 185 stools were sampled from 45 different public gardens, places, sidewalks and dog relaxing areas (1 to 22 samples per area) of Lyon (*n* = 38), France, and neighboring villages (*n* = 7). Samples were taken using e-swabs (bioMérieux, France) which were dipped and turned into individual feces. To decrease the risk of picking multiple stools from the same dog, each area was visited once and only fresh stools, located far away from each other and drastically differing by size, color and consistency were sampled. ESC- and carbapenem-resistant Enterobacterales were selected on ChromID ESBL and ChromID Carba Smart media (bioMérieux), and identification was performed using Maldi-TOF MS. Among the 45 areas sampled, 22.2% (10/45) were positive for ESBL/AmpC-producing Enterobacterales, of which one was additionally a *bla*_OXA-48_-positive area (Table 1 and Table S1). In total, 12 ESBL/AmpC-producing E. coli (12/185, 6.5%), four OXA-48-positive E. coli and three OXA-48-positive K. pneumoniae were identified. All 19 isolates were short-read sequenced using the NovaSeq6000 technology (Illumina, BioProject accession number PRJNA793783). Quality controls are presented in Table S1.

**TABLE 1:** Characteristics of ESC- and carbapenem-resistant isolates collected in healthy dogs

Strain	Species	Faeces no.	Localization	Sequence type	Phylogeny^*a*^	ESBL	OXA-48	pAmpC
51456	E. coli	21	City center 1	48	A	CTX-M-1		
51457	E. coli	56	Village 1	95	B2	CTX-M-15		
51458	E. coli	57	Village 2	1112	A	CTX-M-1		
51463	E. coli	82	City center 2	14	B2	SHV-12		
52040	E. coli	89	City center 3	12	B2			CMY-2
51464	E. coli	104	City center 4	162	B1	CTX-M-15		
51466	E. coli	118	City center 5	58	B1	CTX-M-1		
52041	E. coli	157	City center 6	131	B2	CTX-M-27		
51459	E. coli	60	City center 7	131	B2	CTX-M-15		
51460	E. coli	61	City center 8	131	B2	CTX-M-15		
51461	E. coli	63	City center 8	131	B2	CTX-M-15		
51462	E. coli	76	City center 8	131	B2	CTX-M-15		
**51467** ^*b*^	E. coli	74	City center 8	1730	B1		OXA-48	
**51468**	E. coli	74	City center 8	162	B1		OXA-48	
**51470**	E. coli	79	City center 8	1248	B1		OXA-48	
**51472**	E. coli	81	City center 8	10	A		OXA-48	
**51474**	K. pneumoniae	74	City center 8	11			OXA-48	DHA-1
51475	K. pneumoniae	79	City center 8	11			OXA-48	DHA-1
51476	K. pneumoniae	81	City center 8	11			OXA-48	DHA-1

a Phylogenetic groups (A, B1, B2 or D) as defined by Doumith et al.

b All isolates were short-read sequenced (Illumina NovaSeq 6000) and isolates in bold were additionally long-read sequenced (Oxford Nanopore).

Among the 12 ESBL/AmpC-producing E. coli, a large diversity of enzymes was found, i.e., CTX-M-15 (*n* = 6), CTX-M-1 (*n* = 3), CTX-M-27 (*n* = 1), SHV-12 (*n* = 1), and CMY-2 (*n* = 1). Eight different STs belonging to phylogroups A, B1 and B2 were identified (5). The combination ST131/CTX-M-15 found in four different feces, of which three were collected in the same location (Table 1). Pairwise SNP distances calculated from core genome alignments generated with the Roary pipeline v.3.13.0 showed that these four isolates were identical (0-1 SNP, Table S1). Three ST131/CTX-M-15 samples were recovered from the same dog relaxing area (Lyon center 8) while the fourth one was collected on a public place (Lyon center 7) located 300 m apart. Even though we cannot entirely exclude that feces from a single dog were sampled twice, we suggest that the three ST131-positive feces from Lyon center 8 originated from different dogs, indicating a local cluster of transmission. The last ST131/CTX-M-15-positive feces collected in Lyon center 7 also belonged to this cluster, possibly originating either from a unique dog visiting the two areas successively, or by two different dogs that have exchanged this clone by close contacts or from a common source. Two E. coli ST162 were also collected from two different samples (Table 1) but differed by 1603 SNPs and did not carry the same beta-lactam resistance genes (*bla*_CTX-M-15_ versus *bla*_OXA-48_) so that an epidemiological link was excluded in that case.

**FIG 1:**
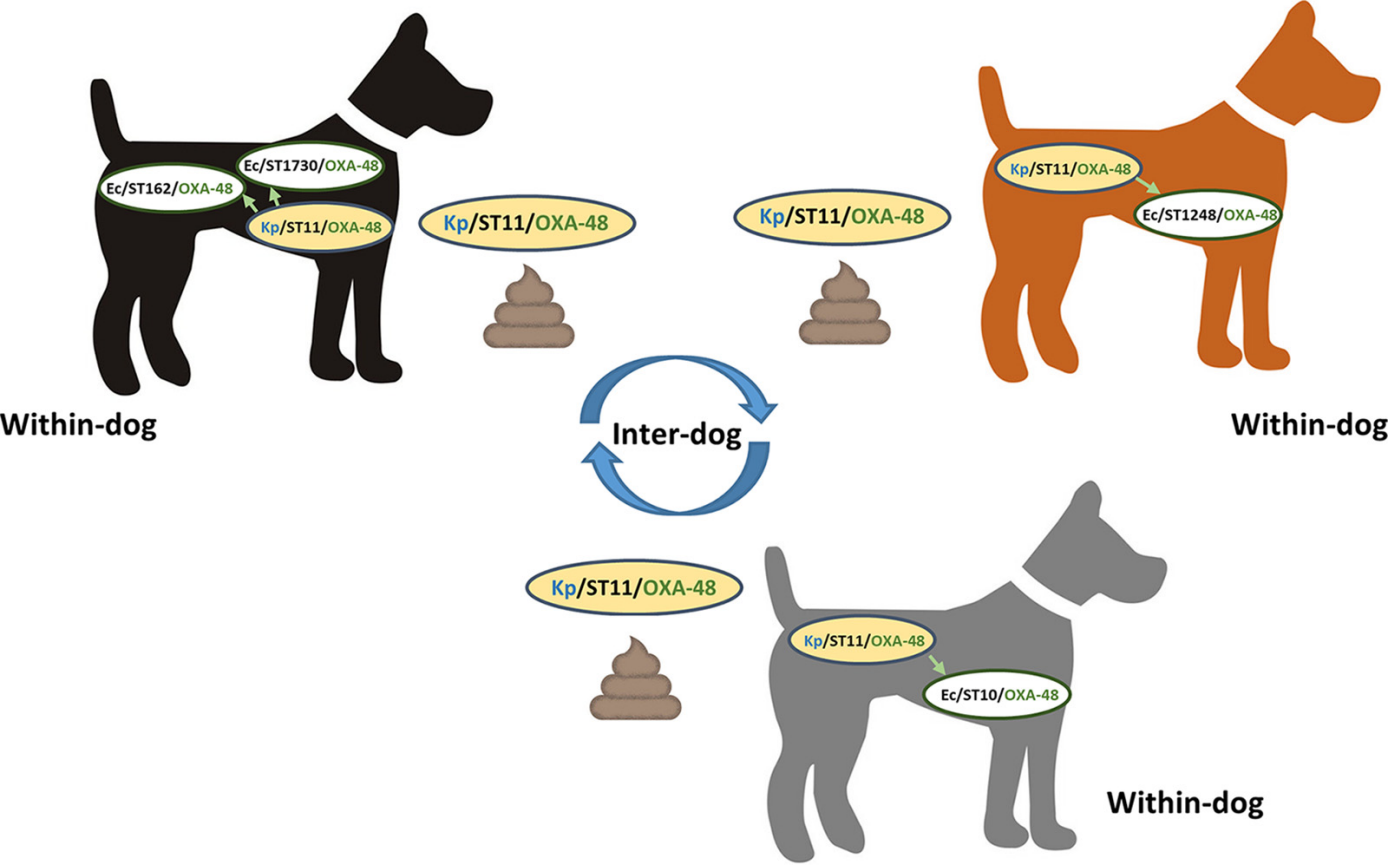
Schematic representation of the *bla*_OXA-48_/IncL plasmid spread within one dog and between dogs.

All seven OXA-48-producing E. coli and K. pneumoniae were collected in the same location (Table 1). They all presented reduced susceptibility to carbapenems, with MICs of 0.75 mg/L for imipenem, 0.19 mg/L for meropenem, and 0.5 mg/L for ertapenem for E. coli isolates, and 0.75 mg/L for imipenem, 0.5 mg/L for meropenem, and 1.5 mg/L for ertapenem for K. pneumoniae. All three OXA-48-producing K. pneumoniae belonged to ST11 and differed by only three SNPs. Considering the habits of owners walking their dog, this situation suggests transmission of the OXA-48 ST11 K. pneumoniae clone between dogs visiting this area, either directly through close contacts or from fecal deposits as a common oral source (Fig. 1). Strikingly, each of the three feces (no. 74, 79, 81 in Table S1) also presented at least one OXA-48 producing E. coli. Sample number 74 carried two different OXA-48-positive E. coli (one ST1730 and one ST162), while feces number 79 and number 81 carried a ST1248 and a ST10 E. coli, respectively. Long-read (MinION, Oxford Nanopore) sequencing of one representative of the ST11 K. pneumoniae (number 51474) and the four OXA-48-positive E. coli (number 51467, 51468, 51470 and 51472) was performed, and data were combined with Illumina sequences using Unicycler (BioProject PRJNA793783). Results showed that the *bla*_OXA-48_ gene was located on IncL plasmids sharing 99.98%–100% identity over the whole 63°589 bp sequence, and zero SNP according to the CSI phylogeny (https://www.genomicepidemiology.org/). Even though independent acquisitions cannot be excluded, these results and the globally low prevalence of OXA-48 in dogs in France rather argue for within-dog *bla*_OXA-48_/IncL transfers from the ST11 K. pneumoniae to commensal E. coli. E. coli ST10, ST1248 and ST1730 displayed no additional antimicrobial resistance genes and were most likely nonpathogenic E. coli isolates naturally residing in the dog’s gut. On the contrary, all three K. pneumoniae and E. coli ST162 were also resistant to tetracyclines, chloramphenicol, trimethoprim-sulfonamides, aminoglycosides, and fluoroquinolones as determined phenotypically (using the disc diffusion method) and genetically (based on WGS analysis).

In conclusion, this study showed that 6.5% of urban dogs were colonized by ESBL-positive Enterobacterales, which is similar to ESBL carriage rates in the French human population (6), and that city parks might be privileged areas of dissemination of MDR bacteria. More importantly, the *bla*_OXA-48_ gene was identified in three feces (3/185, 1.6%), and we witnessed the spread of the *bla*_OXA-48_/IncL plasmid from one K. pneumoniae to commensal E. coli isolates within the dogs’ gut. Considering dog coprophagic behaviors and licking habits as well as the successful spread of the *bla*_OXA-48_/IncL plasmid, such plasmid transfer may explain, at least partly, the persistence of carbapenemases in dogs in the absence of carbapenem use. Consequently, picking up dog feces deposits may contribute to disrupt MDR bacteria and plasmid spread cycles in a One Health perspective.
